# Apoptotic Signaling Pathways in Glioblastoma and Therapeutic Implications

**DOI:** 10.1155/2017/7403747

**Published:** 2017-11-12

**Authors:** Silvia Anahi Valdés-Rives, Diana Casique-Aguirre, Liliana Germán-Castelán, Marco A. Velasco-Velázquez, Aliesha González-Arenas

**Affiliations:** ^1^Departamento de Medicina Genómica y Toxicología Ambiental, Instituto de Investigaciones Biomédicas, Universidad Nacional Autónoma de México, Ciudad de México, Mexico; ^2^Departamento de Farmacología, Facultad de Medicina, Universidad Nacional Autónoma de México, Ciudad de México, Mexico; ^3^Unidad Periférica de Investigación en Biomedicina Translacional, ISSSTE C.M.N. 20 de Noviembre, Facultad de Medicina, Universidad Nacional Autónoma de México, Ciudad de México, Mexico

## Abstract

Glioblastoma multiforme (GBM) is the most hostile type of brain cancer. Its aggressiveness is due to increased invasion, migration, proliferation, angiogenesis, and a decreased apoptosis. In this review, we discuss the role of key regulators of apoptosis in GBM and glioblastoma stem cells. Given their importance in the etiology and pathogenesis of GBM, these signaling molecules may represent potential therapeutic targets.

## 1. Introduction

The most frequent and aggressive human brain tumor is glioblastoma multiforme (GBM), which is a glial cell-derived tumor (glioma) with high malignant potential that has a tendency to invade the surrounding tissue [1]. These tumors arise either from glioma altered cells that facilitate tumor initiation and progression or from glioblastoma stem cells (GSCs) that possess the ability to self-renew and initiate tumor formation [2]. 

GBM originates anywhere in the brain but is mainly located in the cerebral cortex, appearing more frequently in adults between 40 and 60 years old [1]. GBM represent the maximal progression stage of astrocytomas, which are classified according to their histopathological and molecular features into four grades (I–IV). The survival of patients is inversely related to tumor grade [1]. GBM patients have a median survival of just 4.6 months. In optimally treated patients, the median survival increases to 14 months with a 26% two-year survival rate [3, 4]. The classical chemotherapeutic drug used in the treatment of this kind of tumors is Temozolomide, an agent that alkylates DNA at the N-7 or O-6 positions of guanine residues; therefore, it triggers cell death [5].

GBM can arise de novo (primary GBM) or transform from a lower grade astrocytoma (secondary GBM); thus each is characterized by distinct genetic events. GBM de novo frequently has EGFR amplification (34%) and PTEN loss/mutations (24%), while secondary GBM is characterized by* TP53* mutations (65%) and* IDH1* mutations (70%). Despite these differences, both tumors have the same histopathological features [6–8]. Nevertheless, primary GBM are more aggressive and less responsive to treatment compared to secondary ones.

## 2. GBM Intertumor Heterogeneity

A catalog of genomic abnormalities of GBM has been reported through the Cancer Genome Atlas [9], which allowed Verhaak's group to classify GBM into four subtypes: classical, mesenchymal, proneural, and neural [10]. The classical subtype has* EGFR* amplification and loss of* TP53* and* CDKN2A*. It also harbors an* EGFRvIII* mutation constitutively active due to the deletion of exons 2–7 of the EGFR gene. This subtype presents Nestin overexpression and has activated the Notch and SHH pathways [10]. The mesenchymal subtype is associated with poor overall survival and contains* NF1* mutations and loss of* IDH1, PIK3R1,* and* PDGFRA*. It presents overexpression of* MET* and* CHI3L1* and activation of the NF*κ*B pathway [10].

The proneural subgroup has augmented frequency of mutations in* IDH1 *and* TP53* and amplifications of* PDGFRA* and* PIK3CA/PIK3R1*. This group has the highest percentage of younger patients [10]. The neural subtype has elevated levels of neural markers such as* NEFL*, but it has no unique distinguishing alterations from other classes, although elevated rates of* EGFR, TP53,* and* PTEN *mutations are observed [10].

To date, the differences on apoptotic pathways between GBM subtypes have not been clarified. The identification of the mechanisms leading to decreased apoptosis in each subtype might lead to better therapies in the future. For example, the proneural subtype could be dependent on the PI3K/AKT pathway for survival, whereas the mesenchymal subtype could be dependent on the TNF/NF*κ*B pathway. Thus, epidemiological, molecular, and pharmacological studies are required to identify the best therapeutic strategies for each GBM subtype.

## 3. Signals Regulating Apoptosis in GBM

Apoptosis is an essential mechanism by which the homeostatic balance between cell proliferation and cell death is maintained [24, 25]. In apoptosis, cells activate a molecular pathway that leads them to die in case they become damaged and the cell mechanism fails to repair it. This process can be achieved through the activation of two molecular pathways, the extrinsic and the intrinsic pathway. Both pathways lead to the proteolytic activation caspases [24, 25]. These proteases induce cell changes that include chromatin condensation, DNA fragmentation, membrane blebbing, and cell shrinkage [24, 25]. The extrinsic pathway is triggered from outside the cell by proapoptotic ligands that activate cell surface death receptors. After binding to them, the formation of a death-inducing signaling complex (DISC) is induced. This protein complex activates procaspase-8 and procaspase-10 and, eventually, caspase-3 to execute apoptosis [24, 25]. The intrinsic pathway is activated from inside the cell by severe cell stress, such as DNA damage. The latter promotes mitochondrial outer membrane permeabilization and activation of BH3-only proapoptotic B-cell lymphoma 2 (BCL-2) family proteins. This allows the release of proapoptotic proteins, like cytochrome c and second mitochondria-derived activator of caspase (Smac) from the mitochondria into the cytosol. Cytochrome c forms a complex with apoptotic protease-activating factor-1 (Apaf-1) known as the apoptosome to activate caspase-9. This caspase, in turn, activates the effector caspase-3, caspase-6, and caspase-7, which in turn executes apoptosis [24, 25].

**Figure 1:**
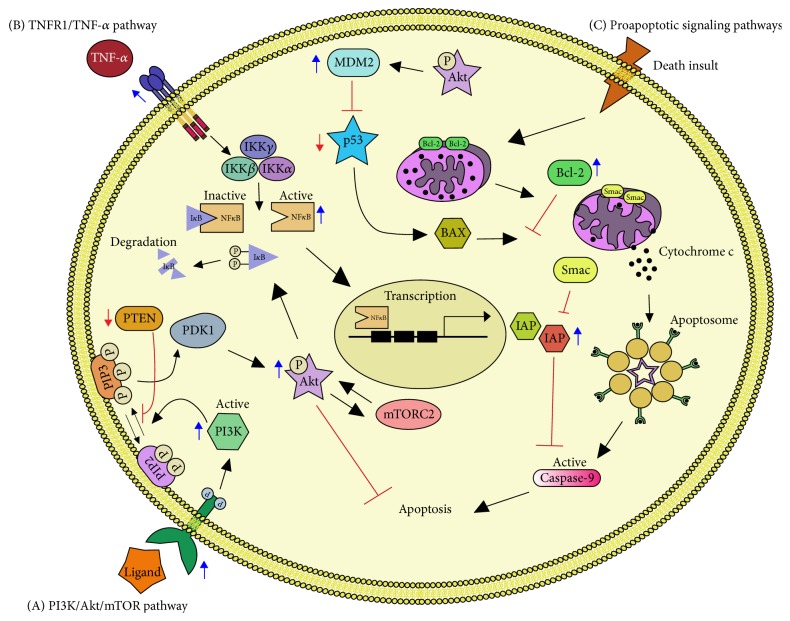
*Apoptotic Pathways in Glioblastoma*. (A) The activation of PI3K ensues by the binding of a ligand to a receptor tyrosine kinase (RTK). The RTK activation by phosphorylation of its intracellular domain activates the catalytic subunit of PI3K; this results in the generation of PIP_3_ from PIP_2_. PIP_3_ activates PDK1 which in turn phosphorylates AKT. AKT phosphorylates many downstream targets, including I*κ*B to induce NF*κ*B activation and mTORC2 and MDM2 to induce cell survival. PTEN antagonizes the PI3K pathway by dephosphorylating the second messenger PIP_3_ to PIP_2_. (B) TNF-*α* is a potent activator of NF*κ*B, which binds to its receptor (TNFR1) enabling the degradation of I*κ*B, by the IKK complex. This allows the translocation of NF*κ*B to the nucleus where it regulates the expression of its target genes such as IAPs, which can directly bind and inhibit caspase-3, caspase-7, and caspase-9. (C) A variety of death* stimuli* can induce the release of cytochrome C from the mitochondria and trigger the formation of the apoptosome with subsequent caspase cascade activation leading to apoptosis. BCL-2 controls the mitochondrial membrane permeability and can inhibit this process, whereas BAX stimulates it. BAX is activated by p53 but p53 is negatively regulated by MDM2. Smac is located within the mitochondrial intermembrane space and enters the cytosol when cells undergo apoptosis to inhibit IAPs. Blue arrows denote overexpression and red arrows denote loss of function.

GMB cells show intrinsic deregulation in apoptotic cell death. Furthermore, antitumor drugs and radiotherapy affect the pathways that control apoptosis in GBM cells, activating prosurvival mechanisms that make the treatments ineffective. Thus, research into the possible enhancement of apoptosis of GBM is a primary goal in the development of new and more effective treatments. In the present review, we summarize the role of apoptosis-controlling intracellular pathways in GBM cells (Figure 1) and discuss their importance as therapeutic targets. Finally, we highlight the differences in apoptosis signaling in the subpopulation of GSCs.

### 3.1. PI3K/AKT/mTOR Pathway Inhibits GBM Apoptosis

PI3K is a kinase that plays a central role in signaling pathways controlling cell survival, proliferation, motility, angiogenesis, and apoptosis [25–27]. The activation of PI3K through its cognate cell surface receptors, such as growth factor receptors, phosphorylates the plasma membrane lipid phosphatidylinositol-4,5-biphosphate (PIP_2_) producing the second messenger phosphatidylinositol-3,4,5-triphosphate (PIP_3_). PIP_3_ induces the accumulation of signaling proteins such as AKT and the phosphoinositide-dependent kinase 1 (PDK1) by direct binding. Association with PIP_3_ at the cell membrane results in AKT phosphorylation by induced-proximity to PDK1 [26]. After its activation, AKT regulates downstream targets like GSK-3*β*, Bad, and mTOR complex to name a few [26, 28].

It has been reported that PI3K/AKT signaling is probably deregulated in 80% of all GBM. The latter is estimated because the majority of tumors have overexpression of EGFRvIII variant and loss of PTEN (see Section 3.2) [10, 29]. Furthermore, there is a direct correlation between AKT activation and the histopathological grade of glioma with 84% of GBM positive to phosphorylated AKT (pAKT), whereas it is scarcely detected in healthy tissue [30]. AKT is the intermediary of PI3K-dependent cell survival responses since its absence abrogates these effects.

In GBM, pAKT induces overexpression of MDM2 protooncogene, which is an important negative regulator of p53 [31–33] and inhibits the apoptosis-inducing protein Bad that is inactive when phosphorylated [34]. Moreover, AKT can indirectly activate mTOR, which is a protein kinase critical for cell proliferation deregulated in GBM [11, 35]. mTOR acts as both a downstream effector and an upstream regulator and its signaling is carried out by two protein complexes known as mTORC1 and mTORC2. mTORC1 regulates cell growth, while mTORC2 phosphorylates AKT at Ser-473 and then further takes part in cell survival, metabolism, proliferation, and cytoskeletal organization [11, 36].

The impact of PI3K/AKT/mTOR pathway in cell survival suggests that its inhibition may lead to increase apoptosis and to be of therapeutic value in GBM [35]. Additionally, its inhibition could increase the cytotoxic potential of the glioma-associated microglia because it polarizes microglial cells towards the M1 phenotype, with cytotoxic activities, and prevents the induction of the M2 that promotes tumor growth [36].

Several dual inhibitors targeting PI3K and mTOR have been developed (Table 1). For example, PI-103 and NVP-BEZ235 induce growth blockage and autophagy induction better than does the mTORC1 inhibitor rapamycin, by blocking the phosphorylation of AKT and mTORC1 target 4EBP1 [11, 35]. However, temsirolimus, a small-molecule inhibitor of mTOR, displays a limited clinical efficacy, since only a subset of patients with high levels of phosphorylated p70s6 kinase in tumor samples were more likely to benefit from treatment. Temsirolimus increased 2.3 months the median survival of GBM patients [14].

At the moment, targeted therapy towards PI3K/AKT/mTOR pathway has not achieved satisfactory results. For further studies, it is important to analyze the reason why some treatments are more beneficial than others taking the following into consideration as possible limiting factors: the number of targets they have, the capacity to cross the blood brain barrier and to reach an optimal concentration in the tumor microenvironment, heterogeneity of GBM, and the activation of alternative signaling pathways.

### 3.2. PTEN Loss in GBM Apoptosis

PTEN is a tumor suppressor gene that antagonizes the PI3K/AKT/mTOR pathway by functioning as a lipid phosphatase. PTEN acts on the lipid signaling intermediate PIP_3_ to convert it into PIP_2_, thereby preventing the activation of AKT [37]. PTEN is a unique phosphatase; there are no PTEN-related proteins that compensate for its loss of function. It has been reported that PTEN is inactivated in GBM. Mutations in PTEN have been associated with the malignant evolution of astrocytic tumors since they are most frequently found in GBM and rarely in lower grades [38, 39]. PTEN mRNA levels are much lower in glioma tissue compared with benign brain tumors and tumor-adjacent normal tissues [40]. The lack of PTEN function is associated with poor survival rather than with tumor initiation in anaplastic astrocytoma and GBM [41]. There is extensive evidence suggesting that the loss of PTEN by mutation, methylation, or deletion leads to a decreased apoptosis [37, 42]. The loss of PTEN is frequent in GBM, and therefore it is difficult to target it for therapy. Recently, a correction of PTEN mutation in GBM cell lines was reported [43]. Using adenoassociated virus-mediated gene edition* PTEN* allele was corrected in two GBM cell lines—42MGBA and T98G. In both cases, the edition resulted in reduced cellular proliferation in an AKT-dependent (42MGBA) and AKT-independent (T98G) manner [43]. Thus, genome editing technologies can be applied to correct genetic mutations in a gain-of-function manner [44, 45]. Whether these strategies are useful for GBM treatment in patients and whether tumor editing is possible in the brain remain to be clarified. 

### 3.3. Role of NF*κ*B in GBM Apoptosis

NF*κ*B [nuclear factor kappa B] is a transcription factor for a large group of genes which are involved in apoptosis, cell adhesion, proliferation, and inflammation [46]. NF*κ*B has 5 subunits: p50, p52, p65, RelB, and c-Rel. These subunits exist in homo- or heterodimers, the most abundant dimer being p65/p50. NF*κ*B is sequestered in the cytoplasm by I*κ*B. This association is rapidly interrupted by diverse signals like cytokines, pathogens, stress signals, and radiation [47]. These signals activate a kinase complex known as I*κ*K. I*κ*K complex is formed of three subunits (*α*, *β*, and *γ*) which phosphorylates and inactivates I*κ*B, which allows the nuclear translocation of NF*κ*B and the subsequent regulation of its target genes including those of Inhibitor of Apoptosis Proteins (IAPs) [47, 48]. Levels of activated NF*κ*B, measured by phosphorylation of the subunits p65/p50, are much higher in GBM compared with that of healthy tissue, and they present a positive correlation with glioma grade [30]. Approximately 96% of the tumors analyzed in two independent studies express activated NF*κ*B [30, 49]. Similarly, it has been found that NF*κ*B is constitutively activated in several human-derived glioblastoma cell lines like U251-MG, U87-MG, and U373 [30].

There are two mechanisms of NF*κ*B activation in GBM: AKT phosphorylation of I*κ*B and TNF-*α*/TNFR1 pathway [30, 49–51]. Tumor necrosis factor-*α* (TNF-*α*) induces the main antiapoptotic activity of NF*κ*B through its death receptors TNFR1 by suppression of caspase-8 activation [50, 52, 53]. The expression of TNFR1 is elevated in GBM compared with the lower grade and scarcely detectable in astrocytomas and healthy brain tissue suggesting a vital role in these tumors [54].

Enhancement of apoptosis by targeting NF*κ*B has had several approaches (Table 1). Bortezomib is a proteasome inhibitor that blocks I*κ*B proteins degradation, among others. It has been evaluated in phase 1 clinical trial either alone or in combination with Temozolomide and radiotherapy [15, 55]. The usage of bortezomib alone showed a partial clinical efficacy although it was not reflected on the overall survival. The combined therapy bortezomib with Temozolomide and radiotherapy was reported to be well tolerated and safe. Currently, a phase 2 trial is active with an overall survival of two years for newly diagnosed GBM undergoing this therapy [15, 55]. BAY 11-7082, an I*κ*K inhibitor, effectively inhibits NF*κ*B which leads to lower chemoresistance, improves sensitivity to photodynamic therapy, and induces senescence [16]. To date, no clinical trials have been reported for this inhibitor. Dehydroxymethylepoxyquinomicin (DHMEQ) is an NF*κ*B inhibitor undergoing preclinical testing [17], where it has shown that inhibition of NF*κ*B activation and its nuclear translocation lead to increased apoptosis [17, 56]. 

NF*κ*B influences multiple cellular processes in normal cells. Therefore, care must be taken when blocking the NF*κ*B pathway to minimize off-target effects and unwanted toxicities. Dual or multitarget therapies may prove more beneficial by targeting several regulators of this pathway, although no studies or drugs have been reported to date.

### 3.4. BCL-2 in GBM Apoptosis

The B-cell lymphoma-2 (BCL-2) is a family of proteins ranging in size from 20 to 37 kDa: BCL-2, BCL-x_L_, MCL-1, BCL-w, BFL-1/A1, BCL-B, BAX, BAK, and BOK [57]. This family participates in cell death and modulates different processes that include apoptosis (BAX, BAK, and BID), necrosis (BAX), and autophagy inhibition (BCL-2, BCL-x_L_, MCL-1, and BCL-w) [57]. The 26 kDa form of BCL-2 protein is localized on the outer mitochondrial membrane, nuclear envelope, and endoplasmic reticulum [58, 59]. This protein is able to regulate the outer mitochondrial membrane permeability of transition pores by blocking proapoptotic proteins like BAX and BAK. Thus, it inhibits the release of cytochrome c from the mitochondria and prevents the formation of the apoptosome, activation of caspases, and eventually cell death [60]. BCL-2 protein is overexpressed in GBM [61] and recurrent tumors [62].* In vitro* studies have shown that the use of antisense constructs or chemical inhibitors, such as ABT-737, is capable of mitigating the antiapoptotic effects of BCL-2 and renders the cells sensitive to cytotoxic treatments (Table 1) [18, 63]. However, GBM has shown resistance to ABT-737 and a similar compound ABT-263 by upregulating MCL-1, another antiapoptotic member of the BCL-2 family. The combined use of ABT-263 and GX15-070, an inhibitor of MCL-1, has shown to effectively reverse this resistance [20]. Preclinical studies seem promising, especially when inhibitors are in combination with additional chemotherapy agents [64]. Once the benefit of using BCL-2 family inhibitors has been established, an obstacle to overcome will be to evaluate if these drugs are able to cross the blood brain barrier, since* in vivo* studies have only been done with subcutaneous tumors of GBM cell lines.

### 3.5. Inhibitor of Apoptosis Proteins (IAPs) Role in GBM

IAPs comprise a family of proteins which have a common domain of 70-amino-acid baculovirus repeats (BIR domains) in their structure. This family of proteins consists of 8 members: NAIP, XIAP, cIAP1, cIAP2, ILP2, livin, survivin, and BRUCE [65, 66]. These proteins can inhibit apoptosis by neutralizing active caspases, through their degradation or by blocking caspase-3, caspase-7, and caspase-9 by binding to their catalytically active pockets [65]. Smac is a protein that promotes cytochrome c release and TNF-*α* dependent apoptosis by direct binding and inhibition of IAPs (XIAP, c-IAP1, and c-IAP2), at the same time inhibiting the TNF-*α*/TNFR antiapoptotic pathway [66].

Within the genomic alterations commonly detected in GBM is the amplification of chromosomal band 11q22, which contains IAP1- and IAP2-encoding genes [67, 68]. Three types of IAPs are widely expressed in glioma cell lines at both mRNA and protein level [69]. They participate in cell survival under apoptotic* stimuli*. This suggests that IAPs may play a role in the resistance of gliomas to apoptosis induced by radio- and chemotherapy [69]. In accordance, the inhibition of IAPs, namely, XIAP and survivin, by antisense oligos sensitizes tumor cells to death [70, 71]. Smac mimetics (e.g., LBW242 and BV6) can also sensitize cancer cells to chemo- and radiotherapy and induce regression of malignant gliomas* in vivo* (Table 1) [21–23, 66]. LBW242, in combination with the PDGFR kinase inhibitor AMN107, triggered apoptosis in human GBM cells* in vitro* and had synergistic effects in mouse models of GBM and primary human GBM neurospheres [21]. In the same fashion, BV6 sensitizes GBM to Temozolomide which induces caspase-8 activation and apoptosis [22].

*In vitro* and* in vivo* data show IAPs as promising targets in anticancer therapy, although the efficacy and tolerability of adverse effects of these agents remain to be determined in clinical trials.

## 4. Apoptosis Control in Glioblastoma Stem Cells (GSCs)

GBM tumors present heterogeneous clonal subpopulations. For example, up to three different molecular subtypes have been found in the same patient, with a prevalence of mesenchymal subtype in males [4, 72, 73]. This clonal divergence can be caused by the genetic instability intrinsic to cancer cells combined with the selective pressure elicited by therapeutic interventions [74]. Furthermore, intratumor heterogeneity is increased by cell plasticity, which allows glioblastoma cells to dedifferentiate to a stem cell-like state [75].

GSCs have functional properties distinct to the rest of glioblastoma tumor cells. They can self-renew and generate progenitor cells, creating a hierarchy consisting of subpopulations of tumorigenic and nontumorigenic cells [76]. GSCs drive GBM growth and promote tumor recurrence and drug resistance [77]. Thus, the presence of GSCs is associated with aggressive tumors and higher mortality rates [77]. Accordingly, the monitoring of the intratumor heterogeneity using GSCs molecular markers in the initial surgery and surgery for recurrent GBM may be important for the most effective management of GBM [78]. Furthermore, GSCs have been pointed as targets for the development of better therapies for this kind of tumors [79].

GCSs express different molecular markers associated with their maintenance, including pluripotency factors such as SOX2, Nanog, and OCT4 [80, 81]. However, GSCs are commonly identified by CD133 or CD44 expression. CD133 is a membrane glycoprotein encoded by prominine-1 gene (PROM1). CD133 is expressed in hematopoietic stem cells, endothelial progenitor cells, and neural stem cells [82, 83], and it is essential for the maintenance of GSCs [80]. CD133 silencing in GSCs reduces proliferation, self-renewal, and tumorigenic capacity. Accordingly, GBM patients with high CD133 levels show poor clinical prognosis [84].

CD44 is a surface receptor that preferentially binds to hyaluronic acid (HA) [85]. In cancer cells, CD44 modulates adhesion, migration, and cell division. CD44 is expressed in GSCs and stem cells from other tumors such as breast and colon carcinomas and leukemias [86–88]. In brain tumors, CD44 expression is associated with increased tumor initiation and progression. In a mouse model of glioma, CD44^+/+^ animals developed significantly more high-grade gliomas and had shorter survival times than did CD44^+/-^ or CD44^−/−^ mice [89]. Accordingly, CD44 showed higher expression in higher-grade brain tumors [90].

Recent evidence suggests that CD133^+^ and CD44^+^ cells represent two different populations of GSCs with different transcriptomic signatures and cell functions. GSCs expressing CD133 display a proneural phenotype, while CD44^+^ GSCs are mesenchymal [91]. Patients with tumor CD44 expression have more aggressive, angiogenic, and radiation-resistant phenotypes but respond better to Temozolomide. In contrast, patients with CD133 expression had a better response to radiotherapy [92]. Moreover, CD133^+^ and CD44^+^ cells can be found simultaneously in xenotransplants generated from patients-derived glioma cells. Hypoxia induces a change from CD44^+^ to CD133^+^, but chemotherapy switches from CD133^+^ to CD44^+^ [78]. Thus, cell plasticity allows the bidirectional conversion between the two phenotypes in response to environmental factors.

### 4.1. PI3K/AKT/mTOR Pathway Also Controls Apoptosis in GSCs

The PI3K/AKT/mTOR signaling cascade promotes proliferation and survival in GSCs. The evidence supporting this idea comes mainly from the effects of inhibitors of the pathway in GSCs. For example, the use of FC85, a dual AKT/mTOR inhibitor, induces apoptosis both in glioblastoma cell lines and in GSC-enriched cultures. Furthermore, the reactivation of p53 with an MDM2 inhibitor enhances the apoptotic effect of FC85 in GSCs [33]. NVP-BEZ235 is an imidazoquinoline that acts as a dual inhibitor of PI3K/mTOR, and it is currently in phase 1 and 2 clinical trials for advanced solid tumors and metastatic cancer (Table 1). NVP-BEZ235 inhibits proliferation and enhances radiosensitivity in a cell line derived from CD133^+^ GSCs. The combination of NVP-BEZ235 and radiation blocks cell proliferation and increases apoptosis. Such effects correlate with the increased expression of the proapoptotic proteins BID, Bax, and caspase-3 as well as with augmented radiation-induced DNA damage [13]. The PI3K pathway is also active in cancer stem cells from prostate [93] and breast [94] tumors, highlighting the role of this pathway in the biology of cancer stem cells.

### 4.2. Apoptosis Signals Specific of GSCs

GSCs are phenotypically and biologically different to the GMB bulk cells. Proteins that are differentially expressed in GSCs can regulate their survival. The examples provided below show that this is the case but also demonstrate that GSCs are sensitive to apoptosis-induction when these molecules are targeted.

Signal Transducer and Activator of Transcription-3 (STAT3) is a transcription factor whose activation plays a crucial role in proliferation, apoptosis-induction, and differentiation of GSCs. STAT-3 knock-down reduces the percentage of CD133^+^ cells and neurosphere-formation in GSC-enriched cultures and decreases tumorigenicity* in vivo* [95, 96]. In human GBM cells grown as neurospheres to increase GSC content, pharmacological inhibition of STAT-3 dimerization decreases the expression of pluripotency, proproliferative, and antiapoptotic genes. The treatment reduces BCL-XL and survivin expression, inducing caspase-3 activation and apoptosis in GSCs [97]. Similarly, inhibition of STAT3 with the natural compound cardamonin (2′,4′-dihydroxy-6′-methoxychalcone) suppresses STAT3 downstream gene expression including BCL-XL, survivin, BCL-2, Mcl-1, and VEGF. Consequently, cardamonin inhibits proliferation and induces apoptosis in CD133^+^ GSCs [98].

The Notch signaling pathway is overactivated in GBM, particularly in those with EGFR amplification (reviewed in [99]). The treatment of GBM neurosphere cultures with *γ*-secretase inhibitors to prevent Notch cleavage reduces the number of CD133^+^ cells and* in vitro* clonogenicity, demonstrating that the Notch pathway is important for stemness maintenance. Blockage of the Notch pathway induces both decreased proliferation and increased apoptosis. The latter effect is associated with an inhibition of AKT- and STAT3-phosphorylation and an increase in the proapoptotic cleaved form of caspase-3 [100]. These data suggest that Notch cross-talk with PI3K/AKT and STAT3 signaling promotes the survival of GSCs.

Finally, the Hedgehog pathway has also been implicated in the control of apoptosis of GSCs. Arsenic trioxide (AT) can directly bind and inhibit GLI proteins [101, 102] which are transcriptional effectors of the Hedgehog pathway. The treatment of glioblastoma neurosphere cells with AT induces apoptosis by increasing caspase-3 cleavage. AT reduces the expression of Hedgehog pathway target genes, such as PTCH1, N-Myc, and GLI2, but also the expression of the Notch pathway target genes HES1, HES5, and HEY1 and that of the pluripotency factor SOX2 [103]. These data suggest that apoptosis-induction by AT may be caused by impairment in multiple pathways controlling stemness.

### 4.3. Role of microRNAs (miRNAs) in GSC Apoptosis

**Figure 2:**
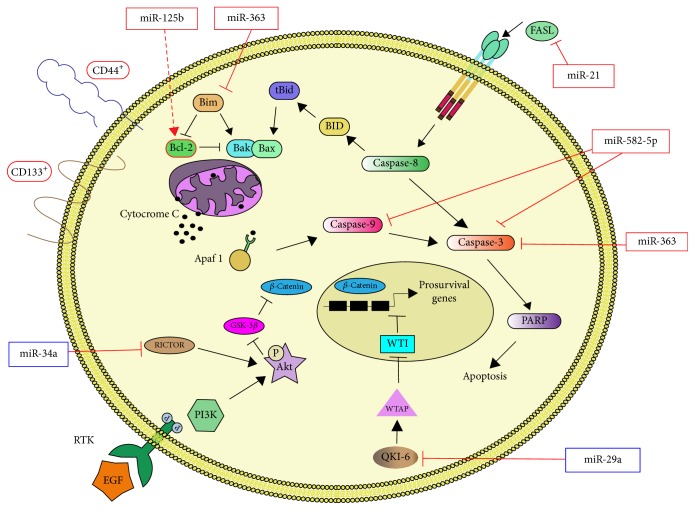
*miRNAs in GSC Apoptosis*. Schematic representation of the interactions between miRNAs and apoptosis signals in GSCs. Red lines represent the findings discussed in text (solid lines for direct and dashed lines for indirect regulation) while black lines represent established apoptotic pathways. Framed miRNAs or proteins indicate whether they are overexpressed (red frame) or underexpressed (blue frame) in GSCs.

At present, studies analyzing the cellular processes regulated by miRNAs in GSCs are scarce. However, as for other CSC, miRNAs seem to play key roles in apoptosis, differentiation, proliferation, migration, and invasion, as well as resistance of GSCs. In this section, we present the linkage of miRNAs to the signaling controlling apoptosis in GSCs (summarized in Figure 2). This information supports the idea that miRNAs can be potential therapeutic targets for the eradication of GSCs, as previously proposed [76, 77].

MiR-125b is overexpressed in CD133^+^ GSCs and its expression correlates with increased resistance to Temozolomide [104, 105]. A reduction in the expression of miR-125b-2 in combination with Temozolomide leads to apoptosis in GSCs by decreasing BCL-2 expression and increasing Bax, cytochrome c, Apaf-1, casapase-3, and poly-ADP-rybose polymerase (PARP) proteins [104]. Furthermore, Bak1 is a direct target of miR-125b in GSCs and its exogenous forced expression restores Temozolomide sensitivity [105]. Similarly, in breast cancer cells, miR-125b confers resistance to paclitaxel through suppression of Bak1 [106].

The expression of miR-21 is also increased in CD133^+^-enriched neurosphere cultures. In those cells, Fas ligand (FASL) is downregulated by miR-21; consequently, they have increased proliferation and reduced apoptosis. The inhibition of miR-21 increases FASL expression and induces apoptosis [107], probably due to the FAS-mediated activation of the caspase cascade. Similarly, miR-582-5p and miR-363 are overexpressed in CD133^+^ human GSCs in comparison to CD133^+^ normal neural stem cells. In GSCs, miR-582-5p and miR-363 target caspase-3, caspase-9, and Bim. Accordingly, the inhibition of the microRNAs restores the expression of targets and leads to decreased cell growth and increased apoptosis [108].

On the other hand, the expression of miR-34a is lower in glioma compared to normal brain tissue and in CD44^+^-enriched glioma cell lines compared with nontumorigenic neural stem cells. MiR-34a targets Rictor, a component of the mTORC2 complex. Thus, ectopic overexpression of miR-34a in GSCs downregulates Rictor, impairing AKT phosphorylation. Reduced activation of AKT, in turn, increases the levels of glycogen synthase kinase-3*β* (GSK-3*β*) and the degradation of *β*-catenin. Consequently *β*-catenin target genes, such as c-Myc and Cyclin D1, are downregulated causing cell cycle arrest and apoptosis [109].

Recently, it has been reported that miR-29a expression is reduced in CD133^+^ GSCs in comparison with that of CD133^−^ nonstem cells. MiR-29a negatively regulates the expression of Quaking gene isoform 6 (QKI-6) by binding to its 3′-UTR. QKI-6 promotes the expression of WTAP [110], which in turn interacts with and inhibits the activity of the transcriptional repressor Wilms Tumor protein 1 (WT1) [111]. Thus, a lower level of miR-29a in GSCs releases the WT1-mediated repression of key prosurvival target genes, such as EGFR [112], and promotes the activation of the PI3K pathway [110]. Consequently, miR29a is a tumor suppressor in GMB and its loss allows GSCs to have continuous activation of pathways controlling both invasion and survival.

## 5. Conclusions

Induction of apoptosis in GBM has showed limited benefits to date. However, clarification of the precise apoptotic mechanism altered in each GBM molecular subtype can develop subtype-specific therapies in the future. Moreover, the development of new therapeutic strategies should consider the presence of GSCs within GBM tumors. GSCs and nonstem GBM cells show common characteristics but also substantial differences. For example, PI3K/AKT/mTOR pathway is deregulated in both subpopulations but the impact of stemness-related pathways or miRNAs in GSCs apoptosis is quite unique.

Thus, in order to increase overall survival of GMB patients, we still require basic and clinical research that focuses on the apoptosis signaling pathways. We foresee that such investigations will uncover new targets for therapeutic intervention.

## Figures and Tables

**Table 1:** Summary of drugs targeting apoptotic pathways in GBM.

Drug	Target	Mechanism of action	STAGE^*∗*^	ID (https://clinicaltrials.gov)	Reference
PI-103	AKT & mTORC1	Prevents phosphorylation of AKT and 4EBP1	PC		[11]
BKM120	PI3K	Direct inhibition of PI3K	Ph I	NCT01339052	[12]
NVP-BEZ235	PI3K and mTOR	ATP-competitive inhibitor of PI3K and mTOR	Ph II	NCT02430363	[13]
Temsirolimus	mTOR	Direct inhibition of mTOR	Ph II	NCT00016328	[14]
Bortezomib	I*κ*B complex	Proteasome inhibitor that blocks I*κ*B degradation	Ph II	NCT00998010	[15]
BAY 11-7082	I*κ*K	Inhibits I*κ*K and prevents NF*κ*B nuclear translocation	PC		[16]
DHMEQ	NF*κ*B	Inhibits NF*κ*B DNA binding	PC		[17]
ABT-737	BCL-2	Direct biding and inhibition of BCL-2	PC		[18]
AT-101	BCL-2, BCL-XL, MCL-1 & BCL-W	BH3 mimetic and BCL-2 family inhibitor	Ph II	NCT00540722	[19]
ABT-263/GX15-070	BCL-2 & MCL-1	Direct biding and inhibition of BCL-2 and MCL-1	PC		[20]
LBW242/BV6	XIAP, c-IAP1, & c-IAP2	Smac mimetic	PC		[21–23]

^*∗*^PC: preclinical; Ph I: phase 1 clinical trial; Ph II: phase 2 clinical trial.
